# Do Colleges Perform the Same Following Developmental Education Reform? The Case of Florida’s Senate Bill 1720

**DOI:** 10.1007/s11162-021-09641-z

**Published:** 2021-06-01

**Authors:** Xinye Hu, Shouping Hu

**Affiliations:** Department of Educational Leadership and Policy Studies, Florida State University, Tallahassee, USA

**Keywords:** Community colleges, Developmental education, Policy implementation, Institutional performance, Student success.

## Abstract

Developmental education (DE) reform took place among the 28 Florida College System (FCS) institutions in 2014. In this study, we examine how cohort-based passing rates in college-level English and math courses changed at different colleges for pre- and post-policy period and explore what institutional characteristics were related with various institutional trajectories of cohort-based course passing rates in the post-policy period. Employing longitudinal data analysis, we found that colleges performed similarly regarding cohort-based passing rates in both college-level English and combined math courses before DE reform and had a similar elevation in the cohort-based English course passing rates when DE reform took place in 2014. However, colleges experienced different change patterns in the years following DE reform. Specifically, colleges located in rural areas and with more White students experienced relatively lower college-level English passing rates in the post-policy period than their counterparts. Different colleges had slight differences in the trajectory of college-level math passing rates by cohort after SB 1720 in 2014, but institutional characteristics in this study did not adequately capture inter-institutional differences.

## Background and Context

Developmental education (DE) courses, or remedial courses, have been part of American postsecondary education for a long time. These courses are traditionally considered as a way to provide opportunities for students who are either less prepared for college courses or have left the postsecondary education system for a period of time to catch up with college-level education. In public two-year colleges, over half of the students enrolled in at least one DE course in 2015 (Schak et al., 2017). In Florida, around 70% of the first-time-in-college (FTIC) students who enrolled in the Florida College System (FCS) colleges in 2009/10 needed at least one DE course (Underhill, 2013), and students paid about $61 million for remedial education courses, as estimated for the 2013/14 school year (Jimenez et al., 2016).

While DE courses are intended to improve students’ academic knowledge and studying skills for college-level courses, it takes students extra time and money to take those courses. Many students in two-year colleges never complete their DE courses. Only 31% of remedial completers are transferred to four-year institutions within six years based on nationally representative data from the 2004-2009 Beginning Postsecondary Students Longitudinal Study (BPS:04/09) (Chen, 2016). According to a report from Complete College America (CCA, 2012), among the 2016 cohort students who enrolled in remedial education, 42% and 27% failed to complete their associated math and English gateway courses in two-year institutions. Simply speaking, DE courses seem to contribute little to student success in college-level courses.

Issues in DE attracted the attention of policymakers and legislators, and over half of the states in the U.S. passed at least one type of legislation to reform DE between 1995 and 2015 (Turk et al., 2015). Legislation in Arkansas and Connecticut focuses on improving DE placement accuracy, while policies in Colorado, Indiana, and Tennessee emphasize expatiating the DE sequences. In Florida, the DE reform legislation – Senate Bill 1720 has been implemented since 2014, aims to accelerate DE courses and increase student academic progression by promoting student self-choices.

In Florida, before SB 1720, colleges placed students into different level courses based on standardized test scores, such as SAT, ACT, and PERT scores. Students who scored below a placement cut-off point were required to take and pass remedial courses before enrolling in gateway and college-level courses. After SB 1720, the placement test is optional for exempt students. The legislation identified exempt students as those who attended a Florida public high school in 2003/04 or later as a ninth-grader and earned a standard high school diploma or became active-duty military personnel. Exempt students don’t have to take placement tests and cannot be required to take DE courses. They can take gateway and other college-level courses directly. Non-exempt students still follow the traditional DE process in terms of taking placement tests and DE courses.

For students who need DE courses, colleges shall deliver DE courses through different instructional strategies such as modularized, compressed, contextualized, and co-requisite instructions to accelerate student progression. The legislation also requires enhancement in advising and student support services to meet individual student needs better. FCS institutions tend to advise the incoming student regarding course enrollment based on student success data like grade point average (GPA), course-taking experiences in high school, work history, military experience, participation in juried competitions, career interests, degree major declaration, as well as standard placement test score (if available for individual students). Additionally, each institution must develop a plan to implement SB 1720.

It was a drastic departure from other DE reforms at the time when SB 1720 was passed in 2013 and implemented in 2014 in the FCS institutions (Hu, 2015). However, in the past a few years, many other states have taken similar approaches in reforming DE, including North Carolina and California. In particular, California’s AB 705 has a lot of resemblance to Florida’s SB 1720 in DE reform. Some other states have been considering similar moves in the past a few years. Thus, findings from studies on the impacts of SB 1720 in Florida would have implications for students in the state and across the country as DE reform has been one of the priorities in state postsecondary education reform.

## Purposes and Research Questions

### Purposes

Previous studies on Florida's DE reform examined the legislation's influences on overall student outcomes. Comparing the student cohort before and after SB 1720, students were more likely to take and pass introductory college-level courses in the first year of policy implementation: the likelihood of enrolling in introductory college-level courses increased by 11.9 percentage points in English Composition 1 and 13.5 percentage points in Intermediate Algebra from Fall 2013 to fall 2014 (Park et al., 2016). Students’ passing rate in college-level courses by cohort (measured as a percentage of course-passing students over their entire cohort) increased by 5.4 percentage points in English Composition 1 and 3.8 percentage points in Intermediate Algebra from Fall 2013 to Fall 2014 (Park et al., 2016). The legislation also narrowed the gap of study outcomes between minority students (Black and Hispanic students) and White students in the post-policy period. Park et al. (2018) found higher increasing rates of enrolling, cohort-based passing, and credit accumulation in college-level courses for Black and Hispanic students than White students at the end of the first academic year after the policy implementation. Researchers also compared student success on average in the three-year pre- and three-year post-policy period (2011–2013 vs. 2014–2016) and found that FTIC students attempted 0.42 more credits in college-level courses and earned 0.40 more credits in these courses in the post-policy period by the end of the first academic year (Hu et al., 2019).

Those studies are important in understanding the overall impacts of SB 1720 on student success statewide. However, the methods using institution fixed effect modeling strategy have an implicit assumption that SB 1720 would have uniform effects across all FCS colleges. In reality, colleges implement SB 1720 with different institutional characteristics, resources, and logics, and treating various institutions as a fixed effect could mask possible institutional differences in student outcomes under the state-wide policy. Thus, it is instructive to examine whether different colleges performed differently over time in student outcomes. Moreover, previous studies comparing overall outcomes before and after policy, this study treats the policy implementation time more flexible: we hypothesize that institutional performances can change year to year after the legislation to track the change pattern over time across institutions. Thus, the present study has two purposes: one is to examine the longitudinal change patterns of the 28 college's performances before and after the DE reform, and the other is to investigate how the institutional characteristics interacted with the DE reform and were related to different performance at different colleges.

### Research Questions

The intent of the legislation was to help students get their educational credentials faster by accelerating students' enrollment and passing college-level courses. The first-year academic performance had a substantial effect on students' three-year persistence and transferring, among other predictors like gender, race, and SES (Allen et al., 2008). Therefore, students' passing rate in the first college-level and the degree-required course is an important indicator showing institutions' performance changes over time. As a college outcome benchmark, this study focuses on college-level courses' success of FTIC students by the end of the first academic year.

According to the Florida Administrative Code (F.A.C.) rule, English Composition 1 is the first degree-credit-bearing English course for all meta-majors. Therefore, English Composition 1 is one of the college-level courses focused on in this study. The college-level math courses are more complicated than the English course since there are various math pathways and course requirements for different meta-majors. The gateway math course was Intermediate Algebra (MAT 1033)—a prerequisite math course for any college-level math course at most colleges in the pre-policy periods. However, it is a prerequisite for the STEM majors’ college-level math courses after the DE reform. Non-STEM students can bypass Intermediate Algebra and enroll in college-level math courses associated with their majors. The Intermediate Algebra is not counted as a degree requirement math credit but can be counted as optional degree credit. Considering non-STEM students can bypass the Intermediate Algebra, the college-level courses in this study only focus on college-level math courses that count for degree required math credits, including College Algebra, Liberal Arts Mathematics I, Liberal Arts Mathematics II, and Introduction to Statistics. These math courses are core math courses for different meta-majors.

Using repeated measurement for cohort-based passing rates in English Composition 1 and college-level mathematics courses at different colleges in different policy implementation years, we examine whether colleges performed differently under the legislation and explore what institutional characteristics contributed to the various performance at colleges. Specifically, we ask the following research questions for college-level English (English Composition 1) and combined math courses respectively:

- Do institution-level *English Composition 1* passing rates based on student cohort change overtime in the pre- and post-policy period? (The intra-institution change).
- How do English passing rates based on student cohort change differently by institutional characteristics over time? (The inter-institution differences).
- Do institution-level *combined introductory college-level math* passing rates based on student cohort change overtime in the pre- and post-policy period? (The intra-institution change).
- How does *combined introductory college-level math* passing rates based on student cohort change differently by institutional characteristics over time? (The inter-institution differences).

We begin by discussing the literature for the study. Next, we describe the longitudinal data analysis method used to analyze the research questions. Then we present the statistic description and longitudinal model results. We conclude with a discussion of the significance of these findings and implications for college leadership, faculty members, policymakers, and additional research.

## Literature Review

Individual colleges responded to policies at the state level differently (Levin et al., 2018). The 28 FCS colleges have different locations, student body size, and student composition. With heterogeneity among community colleges in market, community, and corporation (Thornton et al., 2012), some might adapt to SB 1720 better than others. As a result, college performances and activities could be diverse under the same state policy. Previous works on Florida's DE reform have pointed out that institutional characteristics could be related to policy implementation and contribute to student success (Brower et al., 2017; Nix et al., 2019; Park et al., 2016).

Studies show a large gap between institutions in rural and urban areas regarding college graduate numbers in the United States (Adelman, 2002; Gibbs, 2003, 2005). Theorists postulated that college locations like urbanicity affect student outcomes as an institutional level demographic (Sparks & Núñez, 2014). In our study, colleges located in different areas responded to SB 1720 with various resources and student population. While the policy encourages institutions to provide DE courses using technology, rural college faculty members expressed concern about delivering software- and online-based DE courses, student advising, and tutoring services (Nix et al., 2019). Faculty members in rural colleges also said that their students are limited to access the Internet connection and reliable computers (Nix et al., 2019). From this perspective, colleges located in different areas could have different performance patterns when implementing SB 1720. While the divide between rural and non-rural colleges has been identified from students' and faculty members' perspectives at rural colleges, this study examines how institutional performances changed with college location in the pre- and post-policy periods.

Student body size associating with the campus environment is a structural demographic identified as a factor that influences student outcome (Berger & Milem, 2000; Pike & Kuh, 2005). Under the DE reform, institutions are required to assess students based on multiple dimensions such as their high school transcript, working experience, and career plan. Faculty and staff in smaller colleges might have more opportunities to cooperate with each student; therefore, students in smaller colleges seem more likely to receive accurate advising in course enrollment and adequate assistance in academic study. Based on the IPEDS dataset, the student-faculty ratio is lower in small FCS colleges with student numbers under 5,000, comparing with other colleges with student numbers above 5,000 (16.92 vs 24.3 on average across 2011 to 2016, see Appendix 1); and the small FCS colleges also have higher revenues per FTE students than other colleges (13,726.17 vs 9514.26 on average from 2011 to 2016, see Appendix 1). Colleges with different student body size represent different organization culture under the corporation order and can interact with the state order (SB 1720) differently. Therefore, different implementation results might be observed at colleges with different student body size under the state DE reform.

Institutional compositional demographic characteristics (like gender, race, socioeconomic status, age, and pre-college academic preparation) captures student peer effects and relate to the college environment (Flowers et al., 2001; Whitt et al., 2003). The DE reform in Florida have different influence regarding different student race/ethnicity. The increase in college-level course passing rate by cohort and credit accumulation was greater for Black and Hispanic students following the DE reform in Florida than White students (Hu, Park, et al., 2019). Therefore, colleges with more White students might also show less improvement during the post-policy period.

Research has identified that students from low SES are less likely to attend colleges and less likely to persist or attend graduate schools (Baert & Cockx, 2013; Paulsen & John, 2002; Walpole, 2003). One of the commonly used variables to test the student family status is student free/reduced lunch (FRL) eligibility. FRL eligible students may need additional financial and academic supports at colleges with more FRL eligible students. Data from IPEDS show that the revenue per FTE students is lower in colleges with more FRL eligible students than colleges with less FRL eligible students (10,171.37 vs 10,662.25 on average across 2011 to 2016, see Appendix 1). Additionally, the student-faculty ratio at colleges with more FRL eligible students is higher than colleges with less FRL eligible students (23.80 vs 21.64 on average across 2011 to 2016, see Appendix 1). These data imply that faculty and staff at colleges with more FRL eligible students need to advise and assist more students with less per FTE student revenue. Under the state order of SB 1720, various colleges would show different performances with varying peer and cultural environments.

Academic preparedness is typically regarded as the strongest predictor of student college academic performance and persistence (Pascarella & Terenzini, 2005; Porchea et al., 2010). Numerous empirical studies supported the predictive power of pre-college academic preparedness on student short- and long-term college outcomes (Gifford et al., 2006; Porchea et al., 2010). Park-Gaghan et al. (2019) found that students from the lowest high school academic preparation levels tended to have the most substantial gain from the DE legislation. Following this result, institutions with more academically less prepared students, such as those who ever enrolled in remedial English and who never enrolled in Algebra II in high school, could perform better in college-level classes after the DE reform since those students benefitted more from SB 1720.

Earlier studies in Florida’s DE reform explored different responses in various colleges (Brower et al., 2017). There are different typologies of implementation patterns in the 28 institutions. For example, a large, urban institution with large, diverse, low-income, and immigrant students responded to SB 1720 with an oppositional implementation, and the opposition influenced faculty members to discourage exempt students from taking college-level courses (Brower et al., 2017). In a college with a high proportion of Ph.D.-holding professors and a high student transferring rate to four-year institutions, satisfactory implementations of SB 1720 have been observed (Brower et al., 2017). Ideally, we would like to examine how different implementation patterns affect institutional performance. However, such typology was developed on a limited number of institutions and not available across the 28 FCS institutions.

College leaders’ perceptions regarding SB 1720 implementation have been changing during the post-policy period, suggesting that colleges could have different performances in different policy implementation years. The leaders’ perceptions of the reform process shifted more to institutional staff ownership than state ownership with the implementation time, while there were still various perceptions of the implementation motivations (Mokher et al., 2019).

## Research Design

There are two assumptions behind this study based on previous studies. First, the college performance trajectory in the pre-policy period would be different from the post-policy period in Florida. Second, the patterns of change in institutional performances could differ due to differences in student demographics, institutional size and locations. We use FTIC student college-level course passing rates by cohort from 2011 to 2016 as indicators of institutional performance in this study. Our sample includes 28 FCS institutions. These institutions have implemented the mandatory DE reform (SB 1720) since 2014.

### Data

This study uses institution-level data to examine the overtime impacts of SB 1720 in different FCS colleges. The Integrated Postsecondary Education Data System (IPEDS) provides college’s degree of urbanization (institution location) and institution size for each institution. To avoid the policy’s influence on these college characteristics, this study uses the institutional characteristics records from 2014, when the DE reform policy took place.

The main data source is the Florida K-20 Education Data Warehouse (FL-EDW). This dataset tracks all Florida students from kindergarten to postsecondary education who remain in the state. The FL-EDW dataset contains individual student demographics (race/ethnicity, gender, age, eligibility of free/reduced lunch) as well as high school and college academic records (high school course enrollment and completion condition, college enrollment, college course registration and completion condition). Specifically, we used six cohorts of FTIC student information (three pre-policy cohorts and three post-policy cohorts) to generate aggregated college-level course passing rates by each cohort and institutional demographic composition.

We used institution aggregate data for two reasons, one is policy related and the other is method oriented. For policy makers and institutional leaders, institutional performance and its trajectory has important implications for policy making such as performance-based funding and institutional improvement. For research design consideration, the measure of performance at institutional level could help the data to fit into a hierarchical growth modeling perspective to examine performance trajectories. We acknowledge that analyses using student-level data would provide rich opportunities to examine research questions with advantages of large sample sizes overall and for students of different characteristics. However, student-level data could not directly measure institutional performance and the change of performance over time without being aggregated to institution-level as did in this study. We also recognize that it is important to control for possible changes in student composition over time to accurately examine changes of institutional performance and the role of institutional characteristics. A recent study found that SB 1720 did not result in significant change in student composition in terms of age and race/ethnicity (Hu, Mokher, Zhao, Park-Gaghan, & Hu, 2021).

### Variables

#### Dependent Variables

The FTIC students’ college-level course passing rates by cohort at the end of the first year in each institution were the outcome representing institutional performance over time (each college has a passing rate for each cohort in each year). We focused on two sets of college-level gateway courses for FTIC students: English and the combined math courses.

For each cohort from 2011 to 2016, we tested FTIC students’ cohort passing rates in the 28 colleges by the end of the first academic year. Specifically, we calculated the cohort passing rates as the number of students who passed college-level courses divided by the total number of students in the cohort. For college-level math courses, we accounted students as passing if they passed at least one of the college-level math courses by the end of the first academic year.

#### Independent Variables

The intra-institution differences reflect how institutional performance changed over time. To identify the intra-institutional change over time, we used time variable and policy indicator. The *Time* variable represents the student cohort, which captures the linear slope change (the nature cohort tendency) of student passing rates over the six years. Policy indicators represent the DE policy (SB 1720), which addresses the change in institutional passing rates with the DE reform.

The study tests two types of changes related to the DE reform. The first is the discontinuity change between the pre- and post-policy periods in the initial elevation, which is the cohort-based passing rates’ trajectory on average increase/decrease abruptly upon implementation of SB 1720 in 2014. A binary variable (*Policy*) was used to test such elevation change, with “0” representing the pre-policy period (2011 to 2013), and “1” representing the post-policy period (2014 to 2016). The other change was associated with policy implementation time. Over the years of policy implementation, institutions could have different performances from year to year. The year of policy implementation tests the slope change of trajectories in the years after SB 1720 (Singer et al., 2003). To test the slope change of the passing rates in different years, we created *Implementation year,* which equals to "0" from 2011 to 2013 and equals to "1, 2, 3" in 2014, 2015, and 2016, respectively (Singer et al., 2003). This coding allows the *Implementation* year's coefficient to represent the slope of the post-policy period, which can be different from the pre-policy slope tested by *Time*. *Policy* and *Implementation year* were the independent variables of interest in the level-1 model of multilevel modeling designed for longitudinal data to answer research questions 1a and 2a (How do the gateway course passing rates change among the 28 colleges before and after the DE reform in 2014?).

While *Policy* and *Implementation year* indicate the influence of SB 1720 on institutional performance on average (intra-institution differences), *institutional characteristics* at the level-2 model were used to explain the inter-college differences. The interactions of policy indicators and institutional characteristics allow the study to estimate policy performance in different colleges and capture policy influence conditioned upon each college characteristics. A significant value of interaction indicates that a specific type of college performed better/worse under the DE policy. These tests can help answer research questions 1b and 2b (Do different colleges perform SB 1720 differently?).

Regarding institutional characteristics, we use geographic location (coded as rural vs. non-rural) and the institutional size (coded as small size with less than 5,000 students vs. medium and large size with more than 5,000 students) from the IPEDS dataset. We use an average of each college’s demographic characteristics over six years to represent the college demographic composition. We include the college’s percentage of White, free/reduced lunch eligible students, and students who have completed standard high school math/English as time-invariant indicators representing institutional types. Based on the dummy variable adjustment method (Cohen & Cohen, 1983), we set the missing value in high school math/English to 0, and added another dummy variable (high school missing flag) in the model identifying whether the actual value is missing. This study excluded institutional composition for student age and gender due to the small standard deviation (S.D. < 1.5) of the two indicators among the 28 colleges. (Appendix 2 shows the summary of variable names and descriptions).

### Analytical Methods

Researchers sometimes pool all of the records from multiple subjects and regress the time measures on the outcome using the conventional ordinary least squared (OLS) regression method. However, one fundamental assumption using the OLS regression technique is the independence of observations. Annually repeated measures on the same institution’s course passing rates are not independent—for example, a college’s passing rate in 2014 related to its previous passing rates in 2013, 2012, and 2011. Therefore, the correlation between dependent variables in a longitudinal dataset could violate the assumption of general regression techniques. Additionally, since the OLS regression methods pool all subjects’ measures indiscriminately, the within- and between-institution differences are obscured (Locascio & Atri, 2011). Instead of using the OLS regression method, this study employs multilevel modeling to analyze longitudinal data and answer research questions regarding institutional performance.

The defining feature of longitudinal study design, according to Fitzmaurice et al. (2012), is that "measurements of the response variable are taken on the same individuals at several occasions" (p. 22). The "individuals," also referred to as "subjects," represent the participants or units being studied. For the institutional performance questions, the units are institutions, and the observed variables are college-level courses' cohort-based passing rates by the end of the first year in each institution. The data is balanced in that each institution was measured annually from 2011 to 2016 with incoming first-year students.

The multilevel modeling for longitudinal data specifies a model in which each subject has a unique functional relationship between the outcome and time-related predictors, identifying the correlation between outcomes and time-varying factors in each subject (the intra-difference overtime). The random effects (a group of coefficients, like intercepts and slopes, from different subjects) refer to these functional relationships. In this study, the level-1 model identifies the unique impacts of *Time*, *Policy*, and *Implementation year* on institutional performance at each college and tests whether the residual variation among these relationships is significant. The random effect with insignificant residual variance means that the relationship between time variables (*Time*, *Policy*, and *Implementation year*) and institutional performance was the same for all colleges. Corresponding to the random effect with significant residual variation, the level-2 variables of interest from time-invariant predictors (institutional characteristics) explain the differences between subjects' relationships of time-varying variables and outcome. Since the multilevel modeling for longitudinal data can differentiate intra- and inter-subject differences, this technique is appropriate for answering research questions in this study.

**Fig. 1:**
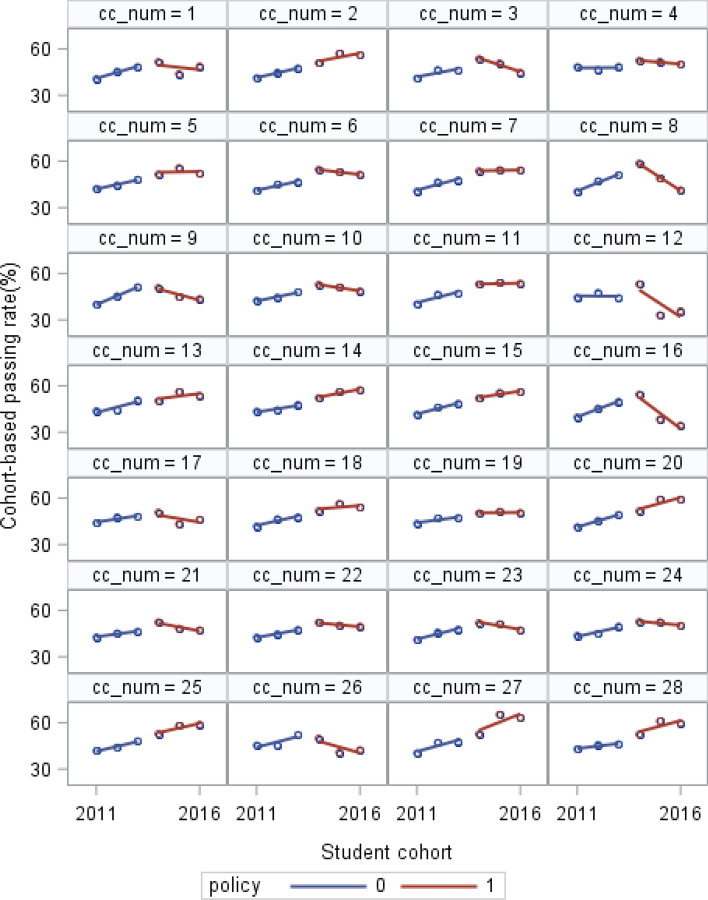
Cohort-Based English Passing Rates from 2011 to 2016 for Each College in the Pre- and Post-policy period

**Fig. 2:**
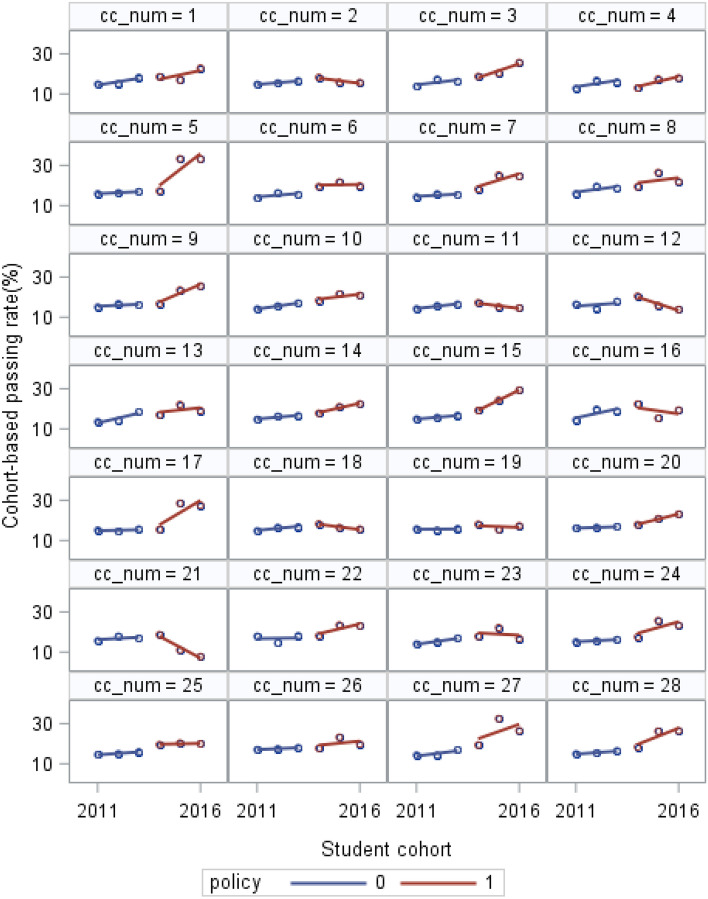
Cohort-Based Math Passing Rates from 2011 to 2016 for Each College in the Pre- and Post-policy period

**Fig. 3:**
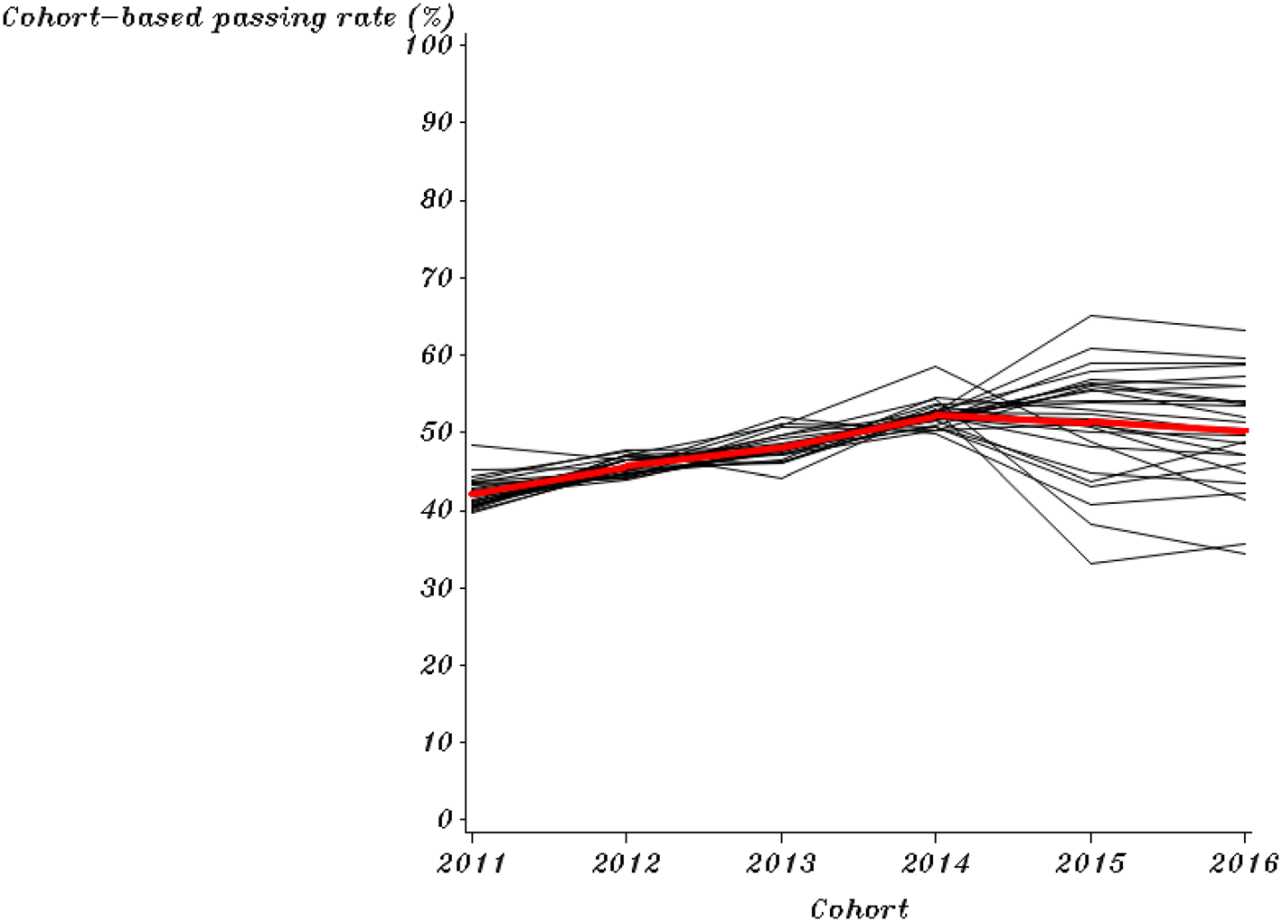
Institutional Trajectories in Cohort-Based English Passing Rates Over 6 Years

**Fig. 4:**
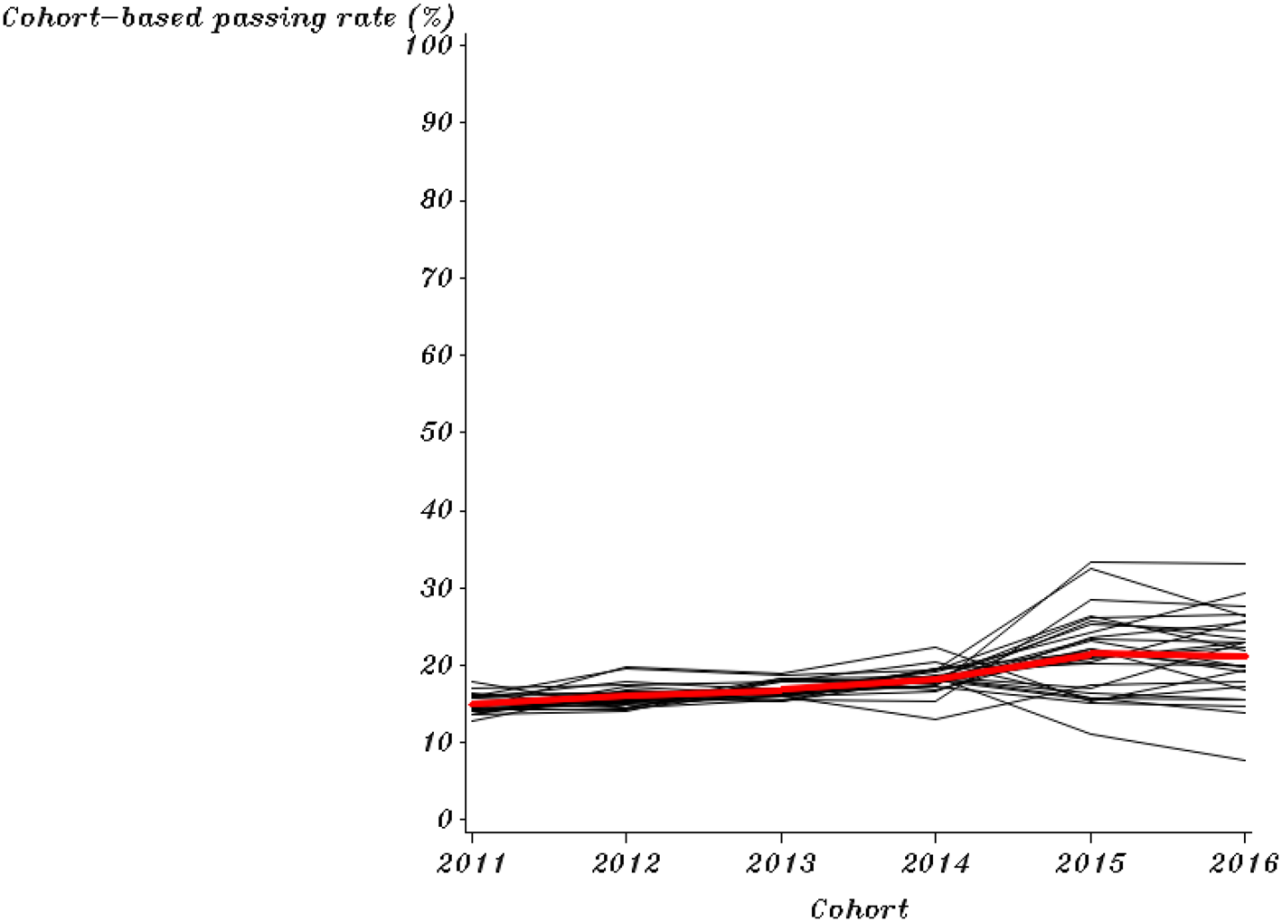
Institutional Trajectories in Cohort-Based Math Passing Rates Over 6 Years

The mean and standard deviations of the outcomes (first-year college-level course passing rates by the cohort of both English and combined math courses) are provided (Table 1). This study provides the trajectory of each institution’s course passing rates over time in an individual plot for each college, which shows the intra-institutional differences in different policy periods (Figs. 1 and 2). A collection of these individual trajectories is also presented, which shows the inter-institutional differences (Figs. 3 and 4). Following the descriptive statistics, this study conducts the unconditional intercept-only model for both English and math courses:

**Table 1:** Mean and Standard Deviation of English and Combined Math Cohort-Based Passing Rates for Each Year (n = 28)

	2011	2012	2013	2014	2015	2016
English						
Mean (%)	42.14	45.64	48.11	52.32	51.40	50.28
S.D	1.92	1.04	1.72	1.70	7.06	6.93
Math						
Mean (%)	14.95	16.03	16.84	18.15	21.49	21.19
S.D	1.11	1.42	0.98	1.62	5.31	5.34

Model 1 s:

Cohort-based English passing rates:

Level-1: $$Y_{ENGij} = b_{0i} + \varepsilon_{ij}$$.

Level-2: $$b_{0i} = \beta_{00} + \zeta_{0i}$$.

where *i* means institutions and *j* means cohort.

Cohort-based combined math passing rates:

Level-1: $$Y_{MATij} = b_{0i} + \varepsilon_{ij}$$.

Level-2: $$b_{0i} = \beta_{00} + \zeta_{0i}$$.

where *i* means institutions and *j* means cohort.

In the level-1 model of Model 1 s, the ${b}_{0i}$ represents the mean of course passing rates by cohort at institution $i$ across six years (2011–2016), and the ${\varepsilon }_{ij}$ represents the residual of cohort $j$ at institution $i$. In the level-2 model of Model 1 s, the ${\beta }_{00}$ represents the grand mean of course passing rate by cohort across colleges, and ${\zeta }_{0i}$ means the residual from college $i$. The residual variance of the intercept (${b}_{0i}$) was not significant (the ${\sigma }_{0}^{2}$ in model 1 is insignificant at α level 0.05 in both English and math models), meaning that there was no statistically significant difference among the means of the course passing rates at the 28 colleges across six years for both English and combined math. Therefore, the following models fixed the intercept.

We then add time-varying variables (*Time*, *Policy*, and *Implementation year*) into the level-1 model (Model 1.1 and Model 1.2 in the Appendix 3). For both English and combined math courses, the residual variances of *Time* and *Policy* were insignificant at α level 0.05 (the ${\sigma }_{1}^{2}$ in model 1.2 and the ${\sigma }_{2}^{2}$ in Model 1.1 for both English and math course). Colleges had a similar *Time* and *Policy* coefficient, meaning that colleges performed similarly before the DE reform and experienced similar performance elevation when DE reform took place in 2014. Therefore, this study only included random slopes for the *Implementation year *since its residual variance was significant at α level 0.05 (the ${\sigma }_{3}^{2}$ in Model 1.2 for both English and math course). The following level-1 models (Model 2 s) were the final level-1 models, with only *Implementation year* being set as a random term. The fixed coefficient ${\beta }_{00}$ represents the estimated course passing rate in 2011, the ${\beta }_{10}$ represents the estimated annual change of course passing rate from 2011 to 2016 if the DE reform did not happen in 2014, and the ${\beta }_{20}$ represents the estimated elevation differential of course passing rate on DE reform implementation. The ${b}_{3i}$ represents the slope differential in pre- and post-policy period regarding the course passing rate by cohort at college $i$, which varied across colleges. The Model 2 s corresponded to Research Questions 1a and 2a—How do the average trajectory of institutions’ *English/combined college-level math* passing rates based on student cohort change over time in the pre- and post-policy period (The intra-institution differences)?

Model 2 s:

Cohort-based English passing rates:

Level-1: $$Y_{Engij} = b_{0i} + b_{1i} time_{ij} + b_{2i} policy_{ij} + b_{3i} imp\_year_{ij} + \varepsilon_{ij}$$.

Level-2: $$b_{00} = \beta_{00} ; b_{1i} = \beta_{10} ; b_{2i} = \beta_{20} ; b_{3i} = \beta_{30} + \zeta_{3i}$$

where *i* means institutions and *j* means cohort.

Cohort-based combined math passing rates:

Level-1: $$Y_{MATij} = b_{0i} + b_{1i} time_{ij} + b_{2i} policy_{ij} + b_{3i} imp\_year_{ij} + \varepsilon_{ij}$$.

Level-2: $$b_{00} = \beta_{00} ; b_{1i} = \beta_{10} ; b_{2i} = \beta_{20} ; b_{3i} = \beta_{30} + \zeta_{3i}$$

where *i* means institutions and *j* means cohort;

The following model includes institutional characteristics to explain the variance in the *Implementation year* and describes the inter-institutional differences in the *Implementation year* for English and combined math passing rates by cohort. In Model 3 s, the ${\beta }_{31}$ to ${\beta }_{36}$ capture the between college difference (by institutional characteristics) regarding the slope of course passing rate in the post-policy period. The Model 3 s correspond to Research Questions 1b and 2b—How do *English/combined college-level math* passing rates based on student cohort change differently by institutional characteristics over time (The inter-institution differences)? The final set of models (Model 3 s) are:

Model 3s:

Cohort-based English passing rates:

Level-1: $$Y_{Engij} = b_{0i} + b_{1i} time_{ij} + b_{2i} policy_{ij} + b_{3i} imp\_year_{ij} + \varepsilon_{ij}$$.

Level-2: $b_{00} = \beta_{00} ; \, b_{1i} = \beta_{10} ; \, b_{2i} = \beta_{20} ; \, b_{3i} = \beta_{30} + \beta_{31} \left( {\% of White} \right)_{i} + \beta_{32} \left( {\% of FRL} \right)_{i} + \beta_{33} \left( {\% of {\text{HS standard English or above}}} \right)_{i} + \beta_{34} \left( {{\text{\% of HS missing flag}}} \right)_{i} + \beta_{35} \left( {{\text{Small}}} \right)_{i} + \beta_{36} \left( {Rural} \right)_{i} + \zeta_{3i}$                                 where *i* means institutions and *j* means cohort;

Cohort-based combined math passing rates:

Level-1: $$Y_{MATij} = b_{0i} + b_{1i} time_{ij} + b_{2i} policy_{ij} + b_{3i} imp\_year_{ij} + \varepsilon_{ij}$$.

Level-2:$$b_{00} = \beta_{00} ; \, b_{1i} = \beta_{10} ; \, b_{2i} = \beta_{20} ; \, b_{3i} = \beta_{30} + \beta_{31} \left( {\% of White} \right)_{i} + \beta_{32} \left( {\% of FRL} \right)_{i} + \beta_{33} \left( {\% of {\text{HS standard math or above}}} \right)_{i} + \beta_{34} \left( {{\text{\% of HS missing flag}}} \right)_{i} + \beta_{35} \left( {{\text{Small}}} \right)_{i} + \beta_{36} \left( {Rural} \right)_{i} + \zeta_{3i}$$.

where *i* means institutions and *j* means cohort;

The final model tests the different slopes in course passing rate after the DE reform in colleges with different characteristics of institutional size, location, and demographic composition; including the percentage of White, free/reduced lunch eligible students, and students who completed high-school standard math/English and above.

## Results

This section first presents descriptive statistics for the institutions’ performance outcomes as measured by annual passing rates of introductory college-level English and combined math after SB 1720. Several graphs are then presented to show trajectories of intra- and inter-institution course passing rates by cohort overtime. Following these descriptive statistics, the multilevel-level modeling results for college-level English and combined math are described.

Table 1 shows the mean and standard deviation of the gateway-course passing rates by cohort for the 28 FCS colleges each year. The means of each year’s English and combined math passing rates for the 28 colleges increased over the six years from fall 2011 to fall 2016. The increasing standard deviation after 2014 shows that the variation in course passing rates by cohort among these colleges increased after implementing SB 1720 in 2014, implying that colleges performed differently after SB 1720.

By checking the normality of both English and combined math passing rates, the cohort-based English passing rates followed normal distribution well, while the cohort-based math passing rates were right-skewed. Therefore, we used log transformation to reduce the skewness of original cohort-based math passing rates (Osborne, 2010).

Descriptive statistics for our independent variables are presented in Table 2 and Table 3. Table 2 shows the means and standard deviations for institutional demographic compositions across the 28 colleges, and Table 3 shows the distribution of institutions by size and location. The categorization of institutions was used in the way to avoid empty cells in any categories.

**Table 2:** Means and standard deviations of institutional demographic composition (n = 168)

	Mean	Minimum	Maximum	S.D
% of White student	43.89	28.48	51.49	5.65
% of FRL student	35.85	30.17	41.32	2.84
% of HS standard English or above	39.11	34.74	43.97	2.27
% of HS standard math or above	33.51	27.95	37.58	2.17
% of HS missing flag	22.10	20.03	24.66	1.33
% of Students aged 25 and older	13.42	11.37	17.42	1.27
% of Female students	52.42	50.09	54.59	1.00

**Table 3:** Frequency Distribution by Institutional Characteristics

	Rural	Non-rural	Total
Small	5	1	6
Medium/large	2	20	22
Total	7	21	28

Figures 1 and 2 illustrate the trajectory of each institution’s cohort-based course passing rates in the pre- and post-policy period in individual plots for English and combined math respectively. According to institutions’ trajectories, cohort-based English passing rates increased at most colleges in the pre-policy period, while a few colleges showed stable patterns (college 4 and college 12). During the post-policy period, English passing rates increased in some colleges (such as college 2, college 27, and college 28), while decreased in some others (such as college 8, college 12, and college16). Regarding the institutional trajectories of cohort-based passing rates in math, most colleges had stable math passing rates with slowly increasing in the pre-policy period. Like the English course, some colleges experienced upward trajectory in the cohort-based math passing rates in the post-policy period (such as college 5, college 9, and college 28), while others experienced downward pattern (such as college 12, college 16, and college 21).

Grouped college trajectories in English and math are presented in Figs. 3 and 4. Each black line represents a college’s passing rate trajectory, showing intra-institutional differences in colleges’ cohort-based passing trends. Before 2014, colleges had similar course passing rates by cohort for English and combined math based on the Figures. After the DE reform in 2014, the trajectory of institutions’ passing rates became more divergent in both English and math. The differences in institutional implementation of SB 1720 can contribute to the diffusion of these 28 trajectories (black lines). Our models investigate institutional characteristics associated with these different trajectories in the college-level course passing rate after SB 1720.

Each graph's red line represents the mean of cohort-based passing rates for the 28 colleges over six years. The red line in the pre-policy period seems to have a lower passing rate on average than in the post-policy period (higher intercept in the post-policy period). Additionally, the red line in the pre- and post-policy periods shows different slopes in English and math. There is a discernable change point in 2014 regarding the slope of institutions' course passing rates by cohort. Our models also examine the trajectory changes in different policy periods.

SB 1720 could influence the average trajectory (the red lines) in two ways. First, institutions’ average passing rates could change abruptly upon SB 1720 implementation in 2014, but the subsequent rate of trajectories had no change (the shift in elevation shows in Fig. 1 and 2); second, institutions’ average passing rates remained stable upon SB 1720 implementation, but the subsequent rate of trajectories changed (the shift in slope shows in Fig. 3 and 4). Based on the passing rate trajectory figures, we chose the linear splines-change model with both elevation and slope change as level-1 models to test the average institutional performance over time.

Based on the results from the unconditional intercept-only model (Model 1 in Tables 4 and 5), there were significant intra-institutional changes over the six years in cohort-based passing rates, on average, across the 28 institutions (variance of residual ${\sigma }_{\varepsilon }^{2}$, is significant at α level 0.05 in both models) for both English and combined math. Our model decision process in the method section addresses that colleges performed similarly in the pre-policy period from 2011 to 2013 and had similar elevation when SB 1720 implemented in 2014 in college-level course passing rates by cohort (no institutional variation regarding the intercept, *Time* and *Policy*). Therefore, models 2 and 3 only contain the random effect of the *Implementation year*. The next sections provide final results from college-level English and combined math passing rates models.

**Table 4:** Results of Longitudinal Models for Change to the Cohort-Based English Passing Rates Data (n = 28)

	Parameter	Model 1	Model 2	Model 3
Fixed Effects				
Intercept	${\beta }_{00}$	48.32***	42.32***	42.32 ***
Time	${\beta }_{10}$		2.98***	2.98***
Policy	${\beta }_{20}$		5.09***	5.09***
Imp_year	${\beta }_{30}$		− 4.00***	− 4.62***
Coefficient of Imp_year (${b}_{3i}$)				
% of White student	${\beta }_{31}$			− 0.30*
% of FRL student	${\beta }_{32}$			− 0.42
% of HS standard English or above	${\beta }_{33}$			0.20
% of HS missing flag	${\beta }_{34}$			− 0.24
Small size	${\beta }_{35}$			0.80
Located in rural	${\beta }_{36}$			− 3.38**
Variance Components				
Level-1:				
Intra-institution	${\sigma }_{\varepsilon }^{2}$	30.37***	4.96***	5.07***
Level-2:				
In intercept	${\sigma }_{0}^{2}$	− 0.5	0	0
In Imp_year	${\sigma }_{3}^{2}$		5.35***	2.97**
Goodness of fit				
− 2LL		1047.3	823.6	805.3
AIC		1053.3	835.6	809.3
BIC		1057.3	843.6	812.0
# of parameters		3	6	12

These models predict cohort-based college-level English passing rates as a function of Time, Policy, and Implementation year (level-1), and combinations of institutional demographics and institutional characteristics (level-2). Statistical significances are indicated by *p < .05, **p < .01, p*** < *.001*

**Table 5:** Results of longitudinal models for change to the cohort-based math passing rates data (n = 28)

	Parameter	Model 1	Model 2	Model 3
Fixed Effects				
Intercept	${\beta }_{00}$	1.248***	1.175***	1.175***
Time	${\beta }_{10}$		0.026***	0.026***
Policy	${\beta }_{20}$		0.015	0.015
Imp_year	${\beta }_{30}$		0.001	− 0.003
Coefficient of Imp_year (${b}_{3i}$)				
% of White student	${\beta }_{31}$			< 0.001
% of FRL student	${\beta }_{32}$			− 0.001
% of HS standard math or above	${\beta }_{33}$			0.001
% of HS missing flag	${\beta }_{34}$			0.008
Small size	${\beta }_{35}$			− 0.021
Located in rural	${\beta }_{36}$			0.007
Variance Components				
Level-1:				
Intra-institution	${\sigma }_{\varepsilon }^{2}$	0.008***	0.002***	0.002***
Level-2:				
In intercept	${\sigma }_{0}^{2}$	< .001	0	0
In Imp_year	${\sigma }_{3}^{2}$		0.001***	0.002**
Goodness of fit				
− 2LL		− 332.7	− 503.8	− 426.7
AIC		− 326.7	− 491.8	− 422.7
BIC		− 322.7	− 483.8	− 420.1
# of parameters		3	6	12

These models predict cohort-based college-level combined math passing rates as a function of Time, Policy, and Implementation year (level-1), and combinations of institutional demographics and institutional characteristics (level-2). The outcome of combined math passing rates by cohort have been transformed using log function. Statistical significances are indicated by *p < .05, **p < .01, ***p < *.001*

### Results for College-level Cohort-Based English Course Passing Rates

Table 4 presents the final model results for over-time English passing rates estimation (Model 2). The average English passing rate for the 28 institutions in 2011 was at 42.32%. The fixed effect of *Time* was significant (${\beta }_{10}$= 2.98, *p* < 0.001), and it represents an expected 2.98% increase in English passing rates by cohort, on average, for the 28 institutions every year (controlling for the DE reform). The annual passing rates of individual colleges increased similarly to the average passing rate as estimated, which is 2.98% (no significant difference between colleges).

Regarding Research Question 1a, there were changes in both the elevation and slope on average English passing rates by cohort from the pre- to post-policy period. The fixed effect of *Policy* was statistically significant (${\beta }_{20}$= 5.09, *p* < 0.001). The average English passing rate by cohort for the 28 colleges increased by 5.09% abruptly in 2014, and all institutions had a similar abrupt increase from pre-SB 1720 (no significant difference between colleges).

The coefficient of *Implementation year* (${\beta }_{30}$) was the mean slope of the 28 colleges' passing rate slopes from 2014 to 2016. The slope of the average passing rates in the post-policy period was significantly different from the pre-policy period. With an abrupt increase in the intercept in 2014 (${\beta }_{20}$= 5.09, *p* < 0.001), the improvement of estimated average cohort-based English passing rate attenuated by 4.00% (${\beta }_{30}$ = -4.00, *p* < 0.001) every additional year from 2014 to 2016 across the 28 colleges. However, the ${\beta }_{30}$ cannot represent the colleges’ overall performance trajectory well. The residual variance of *Implementation year* (${\sigma }_{3}^{2}$) was significant at α level 0.001, suggesting different colleges had significantly different slopes of passing rate from the mean slope (${\beta }_{30}$) during the post-policy period. The large annual decrease at some colleges in cohort-based passing rates after 2014 dragged down the mean passing rate, measured by the coefficient of the *Implementation year* (${\beta }_{30}$). While all colleges experienced an abrupt increase in English passing rates in 2014, some colleges gave back the initial gains in passing rates in the following years after 2014.

Model 2 indicates that when comparing the pre- and post-policy institutional performance, there was a significant increase in English cohort-based passing rates for all colleges in 2014 (the rise in intercept). However, over the extended time of policy implementation, some colleges experienced a decrease while others increased regarding cohort-based passing rates. Model 3 includes institutional characteristics to test how institutional characteristics were related to the different trajectories of institutional performance (Research Question 1b). Model 3 estimates the institutional size, location, and demographic composition (including the percentage of White, free/reduced lunch eligible, and students who completed at least standard high-school English courses) in the level-2 model. Among these independent variables, two variables' effects were statistically significant: geographic location and the percentage of White students on campus.

Based on the level-2 results (Model 3 in Table 4), with an abrupt increase regarding the intercept in 2014 (${\beta }_{20}$= 5.09, *p* < 0.001), the improvement of the cohort-based English passing rate attenuated by 4.62% (${\beta }_{30}$ = -4.62, *p* < 0.001) every additional year from 2014 to 2016, in a typical college with the mean demographic compositions (43.89% White students, 35.85% free/reduced lunch eligible students, 39.11% students completed high-school standard or above English course, and 22.10% students did not have high school course record), controlling for geographic location and institutional size. Colleges located in rural areas and colleges with more White students experienced an offset following the abrupt increasing elevation in the passing rate in 2014 in every additional year. Specifically, with a one percent increase of White students in a typical college with the mean demographic compositions, the institution’s college-level English passing rates by cohort would decrease by 0.30 percentage points (${\beta }_{31}$= -0.30, *p* < 0.05) from the base point of 2014 every additional year after the implementation of SB 1720, controlling for the natural increase from *Time*, other institutional demographic characteristics (free/reduced lunch eligibility and student high-school course completion), institutional location, and institutional size. Similarly, the cohort-based college-level English passing rates in rural areas would be 3.38 percentage points (${\beta }_{36}$= -3.38, *p* < 0.05) lower than in institutions located in non-rural areas, after the implementation of SB 1720 and controlling for other institutional characteristics.

### Results of College-level Combined Math Courses

Table 5 presents results from the final model for the combined math passing rates by cohort estimation (Model 2). Like English course passing rates, the model tested random effects for *Time*, *Policy*, and *Implementation year*. Results showed no college variation regarding the intercept and coefficients for *Time* and *Policy* (the residual variances for the intercept, *Time* and *Policy* were not significant at α level 0.05). Model 2 fixes the intercept, *Time*, and *Policy effects* and keeps the Implementation *year's random effects* as the final level-1 model for cohort-based combined math passing rate analyses (Model 2 in Table 5).

The results show that the average cohort-based math passing rate for the 28 institutions in 2011 was estimated as 14.96% (exponential of ${\beta }_{00}$, 1.175), and all colleges had a similar passing rate in the initial year. The fixed effect of *Time* was significant (${\beta }_{10}$= 0.026, *p* < . 001), and there was no residual variation regarding the coefficients of *Time* (the residual variance of *Time* was not significant at α level 0.05). This result indicated that the average math passing rate by cohort for the 28 institutions increased significantly, by 1.06% (exponential of ${\beta }_{10}$, 0.026) every additional year, controlling for the policy reform, and this annual increase was similar for individual colleges to the average increase (no significant between-college difference).

Regarding Research Question 2a, there was no statistically significant elevation or slope change in combined math passing rates by cohort from pre- to post-policy period, on average. The fixed effect of *Policy* was insignificant (${\beta }_{20}$= 0.015, *p* > 0.05), and there was no residual variation regarding the coefficients of *Policy* (the residual variance in *Policy* is not significant at α level 0.05). This result means that the average cohort-based combined math passing rate across the 28 colleges did not change abruptly in 2014 when the DE reform took place (no elevation change), and individual colleges were similar regarding the insignificant abrupt change of passing-rates (no significant difference between colleges).

Additionally, the coefficient of the *Implementation year* was also insignificant (${\beta }_{30}$= − 0.003, *p* > 0.05), so there was no significant change regarding the predicted slope of average trajectory in the pre- and post-policy period for cohort-based math passing rates among the 28 colleges (no slope change). After estimating the college-level combined-math passing rates by cohort, there were little changes for both elevation and slope regarding the average trajectory across the 28 colleges, comparing the pre- and post-policy periods.

Although the average trajectory remained similar for the pre- and post-policy period, the *Implementation year's* random effect was significant (${\sigma }_{3}^{2}$=0.002, *p* < 0.001), suggesting that colleges performed differently in the years after policy implementation in combined-math courses. Therefore, Model 3 included institutional characteristics to explain inter-institution variations in combined math course passing rates by cohort in the post-policy period. The level-2 model tested how institutional characteristics contribute to the combined math passing rates by cohort (Research Question 2b) after SB 1720.

As with the English research question, institutional characteristics in the level-2 model are institutional size, location, and demographic composition, including the percentage of White students, free/reduced lunch eligible students, students who completed standard math in high school. However, Table 5 (Model 3) shows that none of these variables was significant at α level 0.05. The performances of colleges were different in the years after implementing SB 1720, but the institutional characteristics in this study did not seem to be significant factors contributing to the variances.

## Summary

Regarding the intra-institutional changes over time (Research Questions 1a and 2a), the average trajectory of cohort-based college-level English passing rates changed significantly in elevation and slope across the 28 colleges before and after SB 1720. While the average English passing rate by cohort in the post-policy period was higher than the pre-policy period, the rate of increase reduced each additional year after the policy. However, for college-level combined math courses, the average passing rates had no significant change between the pre- and post-policy periods for either trajectory’s elevation or slope.

Regarding the inter-institutional differences (Research Questions 1b and 2b), the cohort-based passing rates among the 28 colleges were similar before SB 1720 for both college-level English and combined math courses. After SB 1720, both cohort-based college-level English and combined math course passing rates showed significant variations among the 28 colleges, especially the college-level English. Institutions located in rural areas and colleges with more White students had lower cohort-based college-level English passing rates than their counterparts during the post-policy period. Results show significant variation regarding the cohort-based college-level combined math passing rates among the 28 colleges, but the institutional characteristics did not significantly capture such variance.

## Conclusions and Implications

After Florida implemented the comprehensive DE reform (SB 1720) in 2014, several studies investigated the policy (Mokher, et al., 2019; Hu et al. 2019; Park, et al., 2019; Park et al., 2016). Previous studies fixed institutional effects and compared students from the pre- and post-policy periods on average (Hu et al. 2019, Park, et al., 2019). However, some studies identified that different colleges have different policy implementation patterns (Brower et al., 2017), behaviors (Park et al., 2016), and resources (Nix et al., 2019). Therefore, this study tests how different institutions performed differently over time and how institutional characteristics interacted with the DE reform and impacted institutional performance as reflected in cohort-based passing rates in gateway English and combined math courses.

Consistent with findings from previous studies, results in this study show that institutional performance in the gateway English course increased by 5.09 percentage points abruptly in 2014. While previous studies compare student groups with fixed institutional effects in the pre- and post-policy period, this study addresses the influence from the year of policy implementation: the abrupt increase slowed down on average each additional year after policy implementation.

Another focus of this study is the between-college differences in college performance, which has not been studied extensively in previous research. The results show that different institutions performed differently after SB 1720, and such institutional differences, interacting with policy, significantly affect institutional performance. Our results show that colleges in city and suburban areas performed better in the extending years of the DE reform regarding English Composition 1 passing rates by cohort. Nix et al.’s (2019) study also supported this result by demonstrating that colleges serving more rural and low-income students need to find a way to help students in gaining access to and confidence with computers and other technologies.

Colleges with a higher proportion of White students did not seem to perform as well as their counterparts after the DE reform in college-level English. This result is consistent with previous findings that minority students (Black and Hispanic students) benefit more from the DE reform and had higher increase in cohort-based passing rates in college-level courses than White students at the end of the first year after the policy implementation (Hu et al., 2019; Park et al., 2018). One explanation for White students' lower performance after the reform could be that White students might not be highly misplaced in the pre-policy period under the traditional placement model, and the new legislation benefits them less than their minority counterparts. Previous studies pointed out that traditional placement was more likely to assign racial minority students to DE courses (Bailey et al., 2010), which can be a barrier for minority students enrolling in college-level courses. In addition, White students might not be as frequently assigned to DE courses under the traditional placement model in the pre-policy period.

SB 1720 has been less successful in institutions located in rural areas regarding college-level English, and these institutions face more difficulties during the implementation of DE reform. The student-faculty ratios are lower in rural colleges, and the revenue per FTE is higher in rural colleges according to some descriptive statistics (Appendix 1). So the personnel and fiscal resources cannot thoroughly explain the differences in policy success between colleges located in rural and non-rural areas. One possibility could be that institutions located in rural areas face more challenges using technology to offer new DE strategies. As the DE reform suggested, they may need extra technological support to conduct online DE courses and advising systems. Since some new DE courses and other studying resources are computer-based, students at these colleges may also need computer training for technology application under the new DE instructional strategies. State policy should not be one-size-fits-all. Thus, establishing flexible and institution-based policy goals, implementation time, and appropriate supports for different colleges are critical to improving all community college success. Since new DE strategies rely on technology extensively, policymakers may find a way to help rural institutions and their students to access high-speed internet, stable computers, and other necessary technologies.

Studies show that individual student passing rates in Intermediate Algebra from fall 2013 to fall 2014 increased by 3.8 percentage points (Park et al., 2016). However, Intermediate Algebra is not a required math course for a non-STEM college degree in the 28 FCS institutions. The focus of this study is the required college-level math courses bearing degree-required math credits. Three years after implementing SB 1720, however, this study shows no significant difference in the intercepts and slopes in the pre- and post-policy period regarding cohort-based college-level combined math courses’ passing rates trajectory. This finding indicates the importance to further study student performance in math courses to further explore ways to increase student success in college-level math courses. The seemingly non-significant findings regarding institutional performance in combined math courses would be due to the limited statistical power when data were analyzed at institution-level.

Other states, like Colorado (Supplemental Academic Instruction), Indiana (Remedial Co-requisite course), and Tennessee (Co-requisite Remediation Policy) are all redesigning full-semester DE programs to be co-requisite curriculum which is shorter and more efficient (Belfield et al., 2016; Jenkins et al., 2010; Vandal, 2014). While those new DE courses require technical assistance, they may need to realize local inequity regarding the infrastructure. Perhaps other states could help colleges set up essential digital equipment and training programs when implementing computer-based DE/college-level courses.

We also noted that the DE reform contains three major components: the flexibility of course enrollment for exempt students, the new DE instructional strategies, and enhancement of advising and academic support services. Although it is important to study which component in the reform worked effectively, this study did not intend to distinguish each component's impact. Instead, we take the policy implementation time into account and investigate the year-to-year changes in institutional performance, which capture the moving tendency of course passing rates after SB 1720. Additionally, we estimate the between-college differences, emphasizing the heterogeneity of college implementation under a statewide policy. The longitudinal analyses tracked policy implementation and institutional performances show important implications for policy interventions and in evaluating institutional performance. Although the institutional characteristics used in this study do not adequately capture the different patterns in institutional performance, it is important for educational researchers and policy makers to continue to explore what contribute to the differential patterns in institutional performance in broad policy changes as in Florida’s SB 1720.

## Data Availability

One of the datasets that support the findings of this study is available from [Florida K-20 Education Data Warehouse] but restrictions apply to the public availability of these data, because the dataset is used under license for the current study. Another dataset in the current study is available in the [Integrated Postsecondary Education Data System] repository, [https://nces.ed.gov/ipeds].

## Appendix 1

**Table:**

	2011	2012	2013	2014	2015	2016	Mean
Student-faculty ratio by institutional characteristics							
Institution location							
Rural	18.71	18.71	19.57	17.57	17.14	17.43	18.19
Non-rural	24.95	25.33	23.67	24.24	23.90	23.29	24.23
Institution size							
Small	17.33	17.00	18.00	15.83	16.50	16.83	16.92
Medium/large	25.05	25.50	23.91	24.41	23.77	23.18	24.30
% of White student (50-percentile: 44.34)							
< / = 44.34%	25.08	26.31	24.30	25.15	25.15	24.46	25.08
> 44.34%	21.93	21.40	21.20	20.33	19.67	19.53	20.68
% of FRL student (50-percentile: 35.85)							
< / = 35.85%	23.00	22.93	21.71	21.07	21.00	20.29	21.67
> 35.82%	23.79	24.43	23.57	24.07	23.43	23.36	23.78
% of student completed at least standard math course in high school (50-percentile: 33.56%)							
< / = 33.56%	22.36	22.57	21.93	21.43	21.14	20.43	21.64
> 33.56%	24.43	24.79	23.36	23.71	23.29	23.21	23.80
% of student completed at least standard math course in high school (50-percentile: 38.91%)							
< / = 38.91%	22.86	23.71	22.79	23.00	22.36	22.57	22.88
> 38.91%	23.93	23.64	22.50	22.14	22.07	21.07	22.56
Revenues per FTE by Institutional Characteristics							
Institution location							
Rural	12,969.43	11,424.14	12,238.43	13,626.00	14,051.71	13,710.00	13,003.29
Non-rural	9398.00	8552.86	9090.00	9678.43	10,343.48	10,265.14	9554.65
Institution size							
Small	13,442.83	11,800.33	12,746.33	14,428.00	15,053.67	14,885.83	13,726.17
Medium/large	9431.23	8580.77	9094.59	9639.14	10,238.77	10,101.05	9514.26
% of White student (50-percentile: 44.34)							
< / = 44.34%	9483.15	8902.38	9322.46	9795.00	10,195.54	10,134.31	9638.81
> 44.34%	10,990.87	9589.87	10,357.80	11,419.60	12,202.20	11,986.13	11,091.08
% of FRL student (50-percentile: 35.85)							
< / = 35.85%	10,716.07	9451.36	10,062.43	10,846.93	11,550.79	11,345.93	10,662.25
> 35.82%	9865.64	9090.00	9691.79	10,483.71	10,990.29	10,906.79	10,171.37
% of student completed at least standard math course in high school (50-percentile: 33.56)							
< / = 33.56%	10,640.43	9780.57	10,479.29	11,291.71	11,976.21	11,586.14	10,959.06
> 33.56%	9941.29	8760.79	9274.93	10,038.93	10,564.86	10,666.57	9874.56
% of student completed at least standard math course in high school (50-percentile: 38.91)							
< / = 38.91%	10,212.07	9413.93	9969.29	10,914.07	11,296.71	10,999.64	10,467.62
> 38.91%	10,369.64	9127.42	9784.93	10,416.57	11,244.36	11,253.07	10,366.00

## Appendix 2

**Table:** Variable Names and Descriptions

Variable Name	Description
Dependent variables	
English cohort-based passing rate	Calculated as the number of students who passed English Composition 1 by the end of their first academic year divided by the cohort number. Calculated six times for each cohort from 2011 to 2016 in each college
Combined math cohort-based passing rate	Calculated as the number of students who passed at least one of the four college-level math courses by the end of their first academic year, divided by the cohort number. Calculated six times for each cohort from 2011 to 2016 in each college
Independent variables	
Time	Indicates student cohort: 0–5 represents 2011–2016 cohorts
Policy	Indicates SB 1720 implementation status. "0" represents pre-policy period (2011–2013), and "1" represents post-policy period (2014–2016). Tests the elevation differences for average passing rates' trend in pre- and post-policy
Implementation year	Indicates SB 1720 implementation years. “0” represents pre-policy period (2011–2013), “1” represents the first implementation year (2014), “2” represents the second implementation year (2015), “3” represents the third implementation year (2016). Tests the slope differences for average passing rates' trend in pre- and post-policy
% of White students	The percentage of White students. Calculated as the average of six years (2011–2016) for each college. Centered using the mean of the 28 colleges’ % of White students (43.89%)
% of Free/reduced lunch eligible students	The percentage of students who are eligible for free/reduced lunch. Calculated as the average of six years (2011–2016) for each college. Centered using the mean of the 28 colleges’ % of Free/reduced lunch eligible students (35.85%)
% of HS standard English or above	The percentage of students who completed regular or honors English courses and students who completed at least one English course that could account for college credit (dual enrollment, Advanced Placement, or International Baccalaureate). Centered using the mean of the 28 colleges’ % of HS standard English or above (39.11%)
% of HS standard math or above	The percentage of students who completed Algebra II and students who completed at least one more advanced math course, which classified as an “advanced” track in high school. Centered using the mean of the 28 colleges’ % of HS standard English or above (33.51%)
% of HS missing flag	The percentage of students who have a missing value in high school record. Centered using the mean of the 28 colleges’ % of HS missing flag (22.10%)
Location	Indicates institutional geographic location. "-0.5" represents location in city and suburban areas and "0.5" represents location in rural and town areas
Small size	Indicates institutional size. "− 0.5" represents large size with more than 5,000 students, "0.5" represents small size with 5,000 students or less

## Appendix 3

**Table:**

	Parameter	Model 1.1	Model 1.2
Longitudinal Models for Change to the Cohort-Based English Passing Rates Data (n = 28)			
Fixed Effects			
Intercept	${\beta }_{00}$	42.32***	42.32***
Time	${\beta }_{10}$	2.98***	2.98***
Policy	${\beta }_{20}$	5.09***	5.09***
Imp_year	${\beta }_{30}$	− 4.00***	− 4.00***
Coefficient of Imp_year (${b}_{3i}$)			
% of White student	${\beta }_{31}$		
% of FRL student	${\beta }_{32}$		
% of HS standard English or above	${\beta }_{33}$		
% of HS missing flag	${\beta }_{34}$		
Small size	${\beta }_{35}$		
Located in rural	${\beta }_{36}$		
Variance/Covariance Components			
Level-1:			
Intra-institution	${\sigma }_{\varepsilon }^{2}$	5.30***	5.23***
Level-2:			
In intercept	${\sigma }_{0}^{2}$	0	0
In time	${\sigma }_{1}^{2}$	− 0.63**	− 0.20
In policy	${\sigma }_{2}^{2}$	− 0.82	0
In imp_year	${\sigma }_{3}^{2}$	11.41**	8.36**
Time & policy	${\sigma }_{12}$	1.68**	0
Time & imp_year	${\sigma }_{13}$	0.11	− 0.62
Policy & imp_year	${\sigma }_{23}$	− 7.99*	0
Goodness of fit			
− 2LL		799.4	812.4
AIC		821.4	828.4
BIC		836.1	839.1
# of parameters		11	8
Longitudinal Models for Change to the Cohort-Based Math Passing Rates Data (n = 28)			
Fixed Effects			
Intercept	${\beta }_{00}$	1.175***	1.175***
Time	${\beta }_{10}$	0.026***	0.026***
Policy	${\beta }_{20}$	0.015	0.015
Imp_year	${\beta }_{30}$	0.001	0.001
Coefficient of Imp_year (${b}_{3i}$)			
% of White student	${\beta }_{31}$		
% of FRL student	${\beta }_{32}$		
% of HS standard math or above	${\beta }_{33}$		
% of HS missing flag	${\beta }_{34}$		
Small size	${\beta }_{35}$		
Located in rural	${\beta }_{36}$		
Variance/Covariance Components			
Level-1:			
Intra-institution	${\sigma }_{\varepsilon }^{2}$	0.002***	0.002***
Level-2:			
In intercept	${\sigma }_{0}^{2}$	0	0
In time	${\sigma }_{1}^{2}$	< 0.001**	< 0.001
In policy	${\sigma }_{2}^{2}$	− 0.001	0
In imp_year	${\sigma }_{3}^{2}$	0.004**	0.003*
Time & policy	${\sigma }_{12}$	0.001***	0
Time & imp_year	${\sigma }_{13}$	< 0.001	< 0.001
Policy & imp_year	${\sigma }_{23}$	− 0.003*	0
Goodness of fit			
− 2LL		− 529.0	− 511.1
AIC		− 507.0	− 495.1
BIC		− 492.3	− 484.4
# of parameters		11	8

Due to the nature of the algorithms used for maximum likelihood (ML), there are some negative estimates of variance components, negative estimates are constrained to zero according to the recommendation from SAS software website. Statistical significances are indicated by **p* < *0.05, **p* < *0.01, p**** < *0.001.*

## Code Availability

We used STATA/MP 14.2 and SAS 9.4 to complete our work.
